# An Emerging Role of Glucagon-Like Peptide-1 in Preventing Advanced-Glycation-End-Product-Mediated Damages in Diabetes

**DOI:** 10.1155/2013/591056

**Published:** 2013-01-10

**Authors:** Alessandra Puddu, François Mach, Alessio Nencioni, Giorgio Luciano Viviani, Fabrizio Montecucco

**Affiliations:** ^1^Department of Internal Medicine, University of Genoa, Viale Benedetto XV 6, 16132 Genoa, Italy; ^2^Division of Cardiology, Geneva University Hospitals, Faculty of Medicine, Foundation for Medical Researches, Avenue de la Roseraie 64, 1211 Geneva, Switzerland; ^3^First Medical Clinic, Laboratory of Phagocyte Physiopathology and Inflammation, Department of Internal Medicine, University of Genoa, Viale Benedetto XV 6, 16132 Genoa, Italy

## Abstract

Glucagon-like peptide-1 (GLP-1) is a gut hormone produced in the intestinal epithelial endocrine L cells by differential processing of the proglucagon gene. Released in response to the nutrient ingestion, GLP-1 plays an important role in maintaining glucose homeostasis. GLP-1 has been shown to regulate blood glucose levels by stimulating glucose-dependent insulin secretion and inhibiting glucagon secretion, gastric emptying, and food intake. These antidiabetic activities highlight GLP-1 as a potential therapeutic molecule in the clinical management of type 2 diabetes, (a disease characterized by progressive decline of beta-cell function and mass, increased insulin resistance, and final hyperglycemia). Since chronic hyperglycemia contributed to the acceleration of the formation of Advanced Glycation End-Products (AGEs, a heterogeneous group of compounds derived from the nonenzymatic reaction of reducing sugars with free amino groups of proteins implicated in vascular diabetic complications), the administration of GLP-1 might directly counteract diabetes pathophysiological processes (such as pancreatic **β**-cell dysfunction). This paper outlines evidence on the protective role of GLP-1 in preventing the deleterious effects mediated by AGEs in type 2 diabetes.

## 1. Introduction

Glucagon-like peptide-1 (GLP-1) is an incretin hormone that participates to glucose homeostasis. In particular, it has been shown to efficiently lower glucose plasma concentration, improve insulin secretion and resistance, and preserve pancreatic beta-cell function [1, 2]. Due to these proprieties, GLP-1 has been suggested as a promising molecule for the treatment of type 2 diabetes (characterized by hyperglycemia and insulin resistance associated to a progressive deterioration of beta-cell function and mass) [3, 4]. Type 2 diabetes has been also described as a pathophysiological condition accelerating the formation and accumulation of advanced glycation end products (AGEs) that are normally produced with the aging processes [5, 6]. In fact, both repeated acute peaks and chronic hyperglycemia favor the nonenzymatic glycosylation of proteins, lipids, and nucleic acids that after rearrangement, dehydration, and condensation become irreversibly cross-linked, heterogeneous fluorescent derivatives called AGEs. The glycation of these molecules induces alterations in their biological properties as compared with the nonglycated counterparts. Moreover, the binding between AGEs and their receptor (RAGE) provokes oxidative stress generation and inflammatory burst [7]. The pathogenic role of AGEs in microvascular complications of type 2 diabetes is widely investigated and recognized. Recently, some novel detrimental effects of AGEs in type 2 diabetes have been also identified (i) interference by AGEs with the complex molecular pathway of insulin signaling, leading to insulin resistance; (ii) AGE-mediated modification of the insulin molecular structure and function; (iii) AGE-induced decrease insulin secretion and production. Since molecular alterations induced by AGEs are “permanent”, these products have been suggested as key mediators in the “metabolic memory” hypothesis, explaining how diabetic complications are evolving even after glucose control is achieved. In this paper, we will discuss evidence on the protective role of GLP-1 in preventing the deleterious effects of AGEs in type 2 diabetes.

## 2. GLP-1 Production and Secretion

GLP-1 was firstly identified and characterized following the cloning of cDNAs and genes for proglucagon in the early 1980s [8–10]. The major sources of GLP-1 in the body are the intestinal L cells, open-type intestinal epithelial endocrine cells located mainly in the distal ileum and colon, which secrete GLP-1 as a gut hormone [11]. GLP-1 derives from the transcription product of the proglucagon gene which is expressed in the pancreas, intestine, and brain. The proglucagon mRNA is translated into a single-precursor protein that undergoes tissue-specific posttranslational processing operated by prohormone convertase (PC) isoforms, which leads to the synthesis of different proglucagon-derived peptides in the pancreatic *α*-cells and in the intestinal L-cells. Indeed expression of PC 2 in *α* cells leads to synthesis of glucagon, glicentin-related pancreatic peptide, and the major proglucagon fragment, which contains within its sequence both GLP-1 and GLP-2; while expression of PC 1/3 in enteroendocrine L cells results in the production of GLP-1 and GLP-2, as well as glicentin and oxyntomodulin [11]. The prohormone convertase PC1/3 has been localized in intestinal L cells and shown to be both necessary and sufficient for posttranslational processing of proglucagon in the intestine. Indeed, PC1/3 null mice exhibit impaired processing of the precursor with accumulation of intestinal proglucagon coupled to marked decreases in proglucagon processing to glicentin, oxyntomodulin, GLP-1, and GLP-2 [12]. 

The biologically active forms of GLP-1 released in the blood stream are GLP-1(7–36) and GLP-1(7–37), which result from the selective cleavage of the proglucagon molecule [13] and appear equipotent in all biological paradigms studied [14]. In humans, the majority of GLP-1 in the circulation is GLP-1(7–36)NH_2_. The enzyme peptidylglycine *α*-amidating monooxygenase operates the addition of an amide group to GLP-1(1–36) and GLP-1(7–36) to enhance the survival of GLP-1 in plasma [13, 15]. Indeed, the half-life of bioactive GLP-1 in the circulation is less than 2 minutes due to a rapid inactivation by the ubiquitous proteolytic enzyme dipeptidyl peptidase-4 (DPP-4), that cleaves GLP-1 at the N-terminal (GLP-1 9–36) [16, 17].

Accordingly to the position of the L cells (which directly contact the luminal nutrients through their apical surface), GLP-1 secretion is stimulated by a variety of nutrients [18, 19]. Furthermore, the basolateral surface of L cells is located in close proximity to both neurons and the microvasculature of the intestine, which allows the L cell to be affected by both neural and hormonal signals. The mean levels of bioactive GLP-1 in fasting plasma range between 5 and 10 pmol/L in humans and increase approximately 2- to 3-fold after a meal, with the absolute peak values being dependent on both the size and nutrient composition of the meal. Food intake is the primary physiological stimulus to GLP-1 secretion by L cells and results in a biphasic pattern: an initial rapid rise in circulating GLP-1 levels within 15–30 min after a meal, followed by a late minor peak up to 90–120 min [20].

## 3. Intra- and Extrapancreatic Activities of GLP-1

GLP-1 plays multiple roles in metabolic homeostasis following nutrient absorption. One of the first actions identified for GLP-1 was the glucose-dependent stimulation of insulin secretion from islets in rodents, humans, or from islet cell lines [21–24]. GLP-1 induces additional actions in the gastrointestinal tract and central nervous system [25, 26], such as the promotion of insulin biosynthesis, reduction of gluconeogenesis rate, inhibition of glucagon secretion and gastric emptying, and reduction of food intake. The biological effects of GLP-1 are mediated by the binding to its specific receptor (GLP-1R, a specific seven-transmembrane receptor guanine nucleotide-binding protein [G-protein] coupled receptor [GPCR]). GLP-1R was firstly cloned from a rat pancreatic islet library by Thorens in 1992 [27]. The receptor is widely distributed in the pancreatic islets, brain, heart, kidney, and the gastrointestinal tract and mediates the biological actions of GLP-1 in a variety of tissues.

The bioactivities of GLP-1 were investigated mainly in the pancreatic islets. In the pancreas, GLP-1 potentiates glucose-induced insulin secretion improves the function of pancreatic *β*-cells by promoting the genesis and proliferation and by inhibiting apoptotic signals and glucagon secretion from pancreatic *α*-cells, thus resulting in the regulation of glucose homeostasis [28]. GLP-1 synergistically acts with glucose to promote insulin gene transcription, mRNA stability, and biosynthesis, increasing the expression of the transcription factor Pancreas duodenum homeobox 1 (Pdx-1) and the binding of Pdx-1 to the insulin promoter. Furthermore, GLP-1 has been shown to improve glucose sensitivity to glucose-resistant *β* cells [29–31].

In the gastrointestinal system, GLP-1 has been shown to enhance satiety and the feeling of “fullness” delaying gastric emptying, and to inhibit food intake [32–34]. In isolated primary rat hepatocytes and skeletal muscle cells, GLP-1 increases glucose incorporation into glycogen and enhances insulin-stimulated glucose metabolism in adipocytes [35–37]. GLP-1 also inhibits hepatic glucose production and stimulates glucose uptake in fat and muscle and increases glycogen synthase activity and glucose metabolism in skeletal muscle. GLP-1 has been also suggested to induce beneficial effects in the cardiovascular systems, such as some improvements in blood pressure, vascular tone, and myocardial function [38]. There is also increasing evidence from animal models to support a potential role for GLP-1 in neuroprotection, and the increased risk of developing neurodegenerative conditions such as Alzheimer's disease and Parkinson's disease in patients with T2DM suggests that there may be shared underlying mechanisms in these conditions [39].

Impaired GLP-1 physiology is one of several known metabolic deficiencies involved in T2DM. Although controversial data have been reported [40], some studies showed that postprandial levels of intact, biologically active GLP-1 are reduced in obese and type 2 diabetic individuals [41–43]. Despite the reduction of GLP-1 secretion, the glucose-lowering action of GLP-1 are preserved in patients with type 2 diabetes [44, 45], as well as the actions of GLP-1 on inhibition of gastric emptying [46]. On the basis of these findings the augmenting GLP-1 signaling became a useful strategy for treatment of diabetic patients.

The key limitation to the therapeutic use of GLP-1 is represented by its rapid inactivation. To avoid this problem, DPP-4 inhibitors (such as sitagliptin, vidagliptin, or saxagliptin, which stabilize endogenous GLP-1), and more stable exogenous molecules which act as GLP1R agonists (such as the GLP-1 mimetic exenatide, or the human GLP-1 analogue liraglutide) have been used [47–50]. DPP-4 inhibition produces approximately a doubling of the circulating GLP-1 levels, while synthetic agonists mimicking GLP-1 action results in striking elevations of GLP-1 signaling [3, 51–56].

## 4. Formation of AGEs

AGEs are a heterogeneous group of structures formed as both cross- and noncross-linking adducts on proteins, lipids, and nucleic acids [6]. 

In the last century, a nonenzymatic reaction (glycation, underlying AGE formation) was firstly described by Maillard [57]. The Maillard reaction is characterized by the interaction of carbonyl group (aldehyde or ketone) of a reducing sugar with free amino groups of proteins, amino acids, phospholipids, and nucleic acids. The reaction starts with the reversible formation of a Schiff base between a reducing sugar and the amino group of a protein. Under hyperglycemic and/or oxidative stress conditions the relatively unstable Schiff base rearranges to form a more stable Amadori product. Depending on the protein turnover rate and on the glucose concentration, Amadori products undergo further irreversible reactions to form AGEs [58, 59]. The rate and direction of the Maillard reaction may be affected by many others factors, such as the initial pH of the products, the buffer capacity of the system, the temperature and heating time, the moisture content, the nature of the reactants (low molecular weight compounds tend to be more reactive than high molecular weight compounds due to greater steric encumbrance), and the nature of the amino compound (e.g., lysine is more reactive than other amino acids due to the presence of the *ε*-amino group). Besides endogenous formation through the Maillard reaction, AGEs can be also generated from exogenous substances, such as smoke and food. Reactive and toxic glycation products released by combustion of tobacco enter the blood stream and accumulate in the tissues through the lung capillary network [60]. High levels of AGEs are also generated by heating during food preparation and are then absorbed by the intestinal epithelium [61]. Dietary AGEs have a low bioavailability (average 10%), however more than 70% of them were found linked to proteins or incorporated in tissues with consequently injurious impact to vascular and kidney tissues [62].

AGEs are continuously formed and removed in different tissues and body fluids. Formation of AGEs involved modification of long-lived proteins, such as albumin and collagen both in healthy and in diabetic subjects. The mechanism of AGE-mediated cellular damage is a complex process which can be related to chemical modification and cross-linking of tissue proteins, lipids and DNA, and/or mediated by the interaction with specific receptors [63]. The formation and accumulation of AGEs may lead to the modification of intracellular molecules, including most importantly proteins involved in the regulation of gene transcription. Furthermore, the adducts formed as DNA-bound AGEs can affect expression of DNA or even induce permanent mutation. AGEs can also modify extracellular matrix molecules changing signaling between the matrix and the cell, thus causing cellular dysfunction. AGE-modified circulating proteins can bind to AGE receptors, which may have a role in both AGE detoxification and suppression of oxidative stress and inflammation [64, 65]. Therefore, self-maintaining conditions linked to AGE formation would be conceivable as contributing to the so-called “metabolic memory” [66].

## 5. AGEs Activate Injury Pathways in Diabetic Pathophysiology

The key role of AGEs in the onset and evolution of diabetic complications is widely recognized. Several studies reported that the activation of RAGE by AGEs induces the production of inflammatory cytokines and growth factors, which in turn cause vascular pathology [67, 68]. A causal relationship between the markers of early and advanced glycation with diabetic complications has been established in both type 1 and 2 diabetes in basic research and clinical studies [5, 67, 69–71]. 

In the last decade, a direct role of AGEs on pancreatic *β* cell dysfunction in diabetic pathogenesis was also shown [72–76]. The exposure of pancreatic *β* cells to AGEs increased oxidative stress, and contemporarily decreased the already low levels of antioxidant activity [73, 77]. Oxidative stress has been implicated not only in cellular injury, but also in cell death, and the AGEs impaired pancreatic *β*-cell viability was reported in different cell lines [73, 74, 78–80]. Furthermore, AGEs decrease insulin production and release in response to glucose. This events was associated with a decreased expression of PDX-1 (one of the main positive regulators of insulin gene transcription) [72, 81]. Furthermore, we showed that AGEs altered the subcellular localization of transcription factors involved in insulin gene expression. Although the mechanisms of AGE-induced pancreatic *β*-cell dysfunction have to be further clarified, evidence indicates that AGEs interfere with several steps of the insulin-mediated regulation of glucose: AGEs inhibit the production of ATP needed for insulin secretion and decrease the expression of proteins involved in exocytosis of the insulin granules. Moreover, it has been demonstrated that glycation of insulin occurs during diabetes, and that glycated insulin represents a significant proportion of total circulating insulin in type 2 diabetes [82, 83]. Animal studies using isolated muscle and adipose tissue suggest that insulin glycation is associated with a significant compromise of its biological activity. This aspect raises the possibility that insulin glycation might contribute to insulin resistance and glucose intolerance in type 2 diabetes [84, 85]. Besides insulin glycation AGEs may contribute to insulin-resistance at least with two additional mechanisms: (1) evidence that AGE-modified proteins disturb insulin bioactivities in cultured adipocytes and skeletal muscles; (2) impairment of insulin receptor substrate signaling.

These data clearly indicate that the prevention of the synthesis and tissue accumulation of AGE- or oxidative-derived end-products might represent a major advance in the treatment of diabetic complications. Several compounds with putative properties against AGE accumulation have been investigated in both clinical and experimental settings, but in most cases the results were disappointing or inferior to benefits expected [86–90].

## 6. GLP-1 Reduces Expression of RAGE

During the last decade, the promising therapeutic role of GLP-1 to prevent AGE-induced damage was preliminary investigated with encouraging results. Several cell types (such as endothelial cells [91–94], mesangial, neuronal, and pancreatic beta cells [77, 95, 96]) were used *in vitro* at this purpose. 

These studies showed that the interaction between AGEs and RAGE evoked reactive oxygen specie (ROS) generation, eliciting vascular inflammation and thrombosis (conditions described in the diabetic vascular complications). Thus, stopping the vicious cycle triggered by RAGE ligand-RAGE pathway would be helpful to improve the maladaptive effects of glucose in diabetes. Evidence that GLP-1 may be efficient in reducing RAGE expression came out in 2010, when Ishibashi and coworkers demonstrated that GLP-1 dose-dependently decreases constitutive RAGE mRNA levels in an endothelial cell line. In particular, both RAGE mRNA and protein levels were abrogated to about 70% of control cells [91]. Furthermore, authors showed that GLP-1 suppressed the downstream signaling evoked by interaction between AGEs and RAGE and also AGE-induced VCAM-1 expression. One year later, the same group demonstrated in human mesangial cells that GLP-1 decreased RAGE mRNA and protein levels to about 80% of control cells [96]. GLP-1 was able to block the downstream signaling pathway of AGEs-RAGE axis also in these cells, as confirmed by the inhibition of AGE-induced MCP-1 expression. In the same year, Matsui et al. [93] investigated the effects of Vildagliptin, a stable inhibitor of dipeptidyl peptidase-IV, on attenuation of RAGE expression in thoracic aorta of an animal model of type 2 diabetes with obesity, the Otsuka Long-Evans Tokushima Fatty rats (OLETF rats). Quantitative real-time RT-PCR and immunohistochemical analyses revealed that treatment with vildagliptin completely abrogated the increased expression of RAGE mRNA and protein in OLETF rats. Furthermore, vildagliptin blocked the RAGE-downstream pathways in the aorta of OLETF rats, thus preventing the upregulation of two membrane components of NADPH oxidase, gp91phox and p22phox and, subsequently, reducing 8-OHdG level, a marker of oxidative stress generation in the thoracic aorta of OLETF rats. Treatment with Vildagliptin also decreased the accumulation of AGEs in the thoracic aorta of OLETF rats, as revealed by immunohistochemical analyses. Ishibashi et al. demonstrated that Sitagliptin, another stable inhibitor of dipeptidyl peptidase-IV, in combination with 10 pM GLP-1 completely blocked the AGE-induced increase in RAGE mRNA and protein levels in human umbilical vein endothelial cells [92]. Accordingly, in HIT-T15 cells, we demonstrated that GLP-1 counteracted AGE-induced expression of RAGE mRNA, thus attenuating the detrimental effects of AGEs [77]. Interestingly, it has been reported that analogous of cyclic AMP (cAMP) mimicked the effects of GLP-1 on RAGE gene expression in both mesangial and endothelial cells [91, 96]. Since the biological activity of GLP-1 is mainly mediated by the cAMP pathway, the ability of GLP-1 in reducing RAGE expression may be related to a cAMP-dependent pathway activated by GLP-1R.

## 7. GLP-1 Reduces AGE-Induced Oxidative Stress

There is growing body of evidence that AGE-RAGE interaction might provoke oxidative stress generation and inflammation, thereby causing progressive loss of cell function. The restoration of the redox status can lead to the attenuation of the AGE-induced injury and may be achieved through the inhibition of ROS production and/or through the increased expression of antioxidant enzymes. It has been reported that GLP-1 counteracts the AGE-induced ROS generation in many cell cultures [77, 91, 92]. In this context, glutathione might play an important role in the cellular defense against oxidant aggression and in maintaining redox homeostasis. Considering that an increased ratio between the oxidized (GSSG) and the reduced form (GSH) are directly associated with oxidative stress, we found that AGEs increased GSSG to GSH ratio rising the levels of GSSG and reducing in the same time the availability of GSH in a pancreatic beta-cell line [77]. Interestingly, GLP-1 restored the levels of GSH, without affecting GSSG, suggesting that the protective effect of GLP-1 on oxidative stress is mainly related to its ability of increasing antioxidant defense [77]. The same behavior has been observed in neuronal cells [95].

## 8. GLP-1 Prevents AGE-Induced Cell Death

GLP-1 has been shown to prevent AGE-impaired viability in many cell types [77, 94, 95]. This important effect is related to the reduction of oxidative stress and alteration of Bcl-2- and caspase-mediated pathways [73, 77, 94]. Recently, we demonstrated in pancreatic beta cells that GLP-1 was able to prevent AGE-induced necrosis and apoptosis and that this condition is related to a decreased caspase-3 activity [77]. Kimura and coworkers showed that GLP-1 protected neuronal cells against methylglyoxal-induced apoptosis, and that certain signaling pathways (such as PI3 K/Akt/mTOR/GCLc/redox, cyclic AMP (cAMP), and MAPK) have a crucial role in the beneficial effects mediated by GLP-1 [95]. Zhan and coworkers showed that GLP-1 attenuated AGE-mediated proapoptotic activities in endothelial cells by the release of cytochrome *c*, increased expression of the proapoptotic protein Bax, and caspase activation. Taken together, these findings suggest that cAMP might be actively involved in the AGE-triggered signaling pathways. In particular, in human umbilical vein endothelial cells (HUVEC) exposed to AGEs, RAGE might be a critical molecular target that is selectively reduced by the protective pathways activated by GLP1R.

## 9. Conclusion

**Figure 1:**
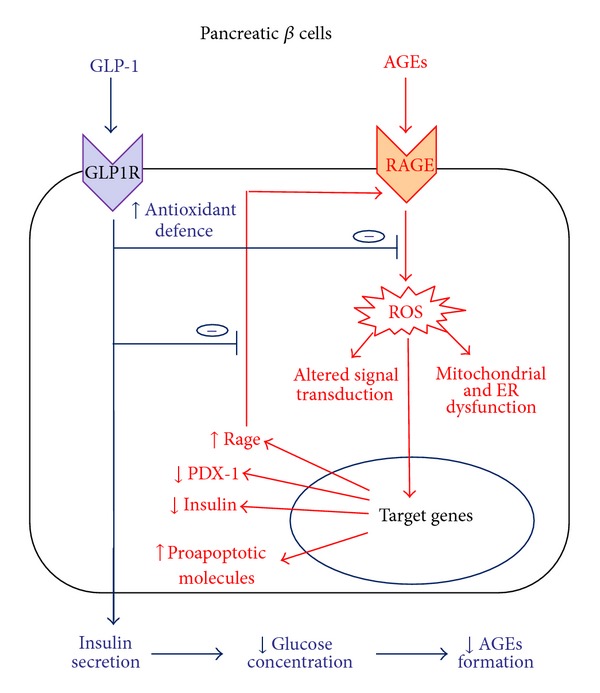
Beneficial effects of GLP-1 in pancreatic beta cells exposed to AGEs. The activation of the AGEs-RAGE axis in pancreatic beta cells increases oxidative stress that causes mitochondrial dysfunction, endoplasmic reticulum stress, and altered signal transduction. These detrimental effects modify gene expression leading to increased expression of RAGE and proapoptotic molecules, and downregulation of proteins involved in insulin gene expression, such as PDX-1, thus causing decreased insulin production and loss of glucose-stimulated insulin secretion (GSIS). Activation of GLP-1 signaling increases antioxidant defense thus counteracting formation of reactive oxygen species (ROS) and expression of RAGE, blocking the positive feedback loop that links RAGE activation with RAGE expression. Furthermore, GLP-1 maintaining pancreatic beta-cell function restores GSIS, thus contributing to reduce plasma glucose concentration and, consequently, formation of new AGEs.

**Figure 2:**
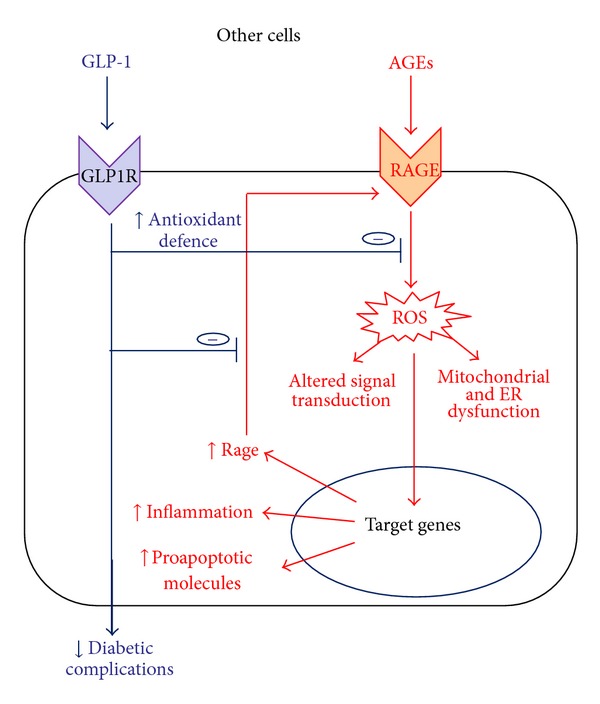
Beneficial effects of GLP-1 in other cells exposed to AGEs. Detrimental effect of AGEs leads to cell dysfunction and, eventually, cell death. GLP-1, counteracting AGEs-induced damage with mechanisms reported in pancreatic beta cells, may contribute to reducing diabetic complications.

Inhibitory treatments targeting the accumulation of AGEs may have beneficial effects on the development/progression of diabetic complications and may protect pancreatic *β* cells from the deleterious effects of the diabetic milieu. In this paper, we highlighted evidence that GLP-1 may counteract AGE-induced damage, preserving the pancreatic *β*-cell function, limiting the disease progression and the development of its vascular complications. These beneficial effects may be attributable not only to the improvement of the glycaemic control (leading to a slowing down in the accumulation of AGEs), but also to the blockage of the AGEs-RAGE axis, which is a pathogenetic mechanism of diabetes and its complications. Despite ameliorating hyperglycaemia, GLP-1 might provide different levels of protection from the deleterious effects of AGEs: (i) by increasing antioxidant defence, thereby reducing cellular stress; (ii) by blocking the positive feedback of AGEs on RAGE expression (Figures 1 and 2).
